# Research of insomnia on traditional Chinese medicine diagnosis and treatment based on machine learning

**DOI:** 10.1186/s13020-020-00409-8

**Published:** 2021-01-06

**Authors:** Yuqi Tang, Zechen Li, Dongdong Yang, Yu Fang, Shanshan Gao, Shan Liang, Tao Liu

**Affiliations:** 1 Department of Neurology, Hospital of Chengdu University of Traditional Chinese Medicine, Chengdu, 610072 China; 2 School of Automation, Chongqing University, Chongqing, 400044 China; 3 Electronic Engineering College, Chengdu University of Information Technology, Chengdu, 610225 China

**Keywords:** TCM, Insomnia, Machine learning, Diagnosis, Association rules, Cluster analysis, Random forest

## Abstract

**Background:**

Insomnia as one of the dominant diseases of traditional Chinese medicine (TCM) has been extensively studied in recent years. To explore the novel approaches of research on TCM diagnosis and treatment, this paper presents a strategy for the research of insomnia based on machine learning.

**Methods:**

First of all, 654 insomnia cases have been collected from an experienced doctor of TCM as sample data. Secondly, in the light of the characteristics of TCM diagnosis and treatment, the contents of research samples have been divided into four parts: the basic information, the four diagnostic methods, the treatment based on syndrome differentiation and the main prescription. And then, these four parts have been analyzed by three analysis methods, including frequency analysis, association rules and hierarchical cluster analysis. Finally, a comprehensive study of the whole four parts has been conducted by random forest.

**Results:**

Researches of the above four parts revealed some essential connections. Simultaneously, based on the algorithm model established by the random forest, the accuracy of predicting the main prescription by the combinations of the four diagnostic methods and the treatment based on syndrome differentiation was 0.85. Furthermore, having been extracted features through applying the random forest, the syndrome differentiation of five zang-organs was proven to be the most significant parameter of the TCM diagnosis and treatment.

**Conclusions:**

The results indicate that the machine learning methods are worthy of being adopted to study the dominant diseases of TCM for exploring the crucial rules of the diagnosis and treatment.

## Background

The application of TCM can be traced back to thousands of years [1]. In spite of the fact that TCM is still regarded as the complementary and alternative therapy in the field of modern medicine, it can hardly be ignored that TCM has attracted widespread attention in recent years due to its unique personalized treatment scheme and the outstanding treatment effect on some dominant diseases [2, 3]. Insomnia is one of the dominant diseases of TCM. It has been proven that TCM has been successfully applied to the treatment of insomnia in the medical field [4, 5]. Compared with the western medicine in the treatment of insomnia, the advantages of TCM treatment are the personalization of diagnosis and treatment ideas, the non-dependence of treatment drugs and the diversity of treatment schemes, etc. Unlike the diagnosis and treatment of the western medicine, which is based on rigorous scientific trials, most of TCM diagnoses are relied on the experience of doctors to get comprehensive and personalized treatment strategies. Consequently, TCM is considered as an empirical medicine as well. Nonetheless, it should be noted that a set of core theories of TCM have been established since the beginning of the TCM development. Subsequently, the core theories of TCM have been developed into the TCM prescription, acupuncture, meridians and other theories [6]. Moreover, in the long-term clinical practice, with the constant deepening of the understanding of the basic theories of TCM, the diagnosis and treatment ideas of TCM have been promoted tremendously, and the diagnosis and treatment standards have achieved an innovation as well [7]. Diagnosis and treatment ideas and treatment strategies are the critical points of the clinical practice. Meanwhile, the medical record data are the embodiment of diagnosis and treatment ideas, thus worth exploring. The medical record of TCM is composed of four parts, including the basic information, the four diagnoses of TCM, the treatment based on syndrome differentiation and the main prescription.

The concept of wholism and the treatment based on syndrome differentiation are the core principles for diagnosing and treating disease of TCM. In recent years, many studies have proposed that the TCM diagnosis and treatment should be integrated and personalized, which was essentially consistent with the core principles of TCM [8]. Syndrome differentiation and disease treatment are two inseparable parts in the process of TCM diagnosis and treatment. Syndrome differentiation is the premise and basis for treatment, and disease treatment is the means and method. The correctness of syndrome differentiation and treatment can be verified by examining the effect of disease treatment. The treatment based on syndrome differentiation is the core principle guiding the clinical work of TCM. In this paper, the four parts of TCM diagnosis and treatment (the basic information, the four diagnoses of TCM, the treatment based on syndrome differentiation and the main prescription) are the specific manifestations of TCM diagnosis and treatment process. The whole diagnosis and treatment process is not only logical, but also indivisible. The diagnosis and treatment of TCM is a whole from the information collection (including basic information and four diagnoses) to the treatment based on syndrome differentiation, and then to the establishment of the main prescription. In the past decades, many efforts have been done to study this process, whereas most researches have only focused on one part of this process. Zhang et al. [9] applied the data mining technology to explore the drug rules of pulmonary fibrosis based on TCM medical records. Yu et al. [10] analyzed the dose data of TCM prescriptions by optimizing the traditional Cheng-Church double clustering algorithm (CC). Liu et al. [11] adopted the data mining method to verify the TCM syndrome patterns of PSCI. These researches have shown some opinions on the diagnosis and treatment process of TCM to some extent. However, their research methods have violated the core principle of integration and personalization of the TCM diagnosis and treatment, resulting that their conclusions can hardly be applied in clinical practice. Therefore, for the sake of reliability and comprehensiveness of the research method adopted in the present paper, the research is carried out logically according to the sequence of TCM diagnosis and treatment, and the whole will be discussed at last.

In recent years, the rapid development of data analysis and artificial intelligence has provided an innovative research direction for the improvement of the clinical diagnosis and treatment technology. In the present paper, the medical record data of insomnia are selected as the research samples. Based on the medical record data, the research method of diagnosis and treatment of insomnia of TCM is emphatically discussed by applying machine learning methods. Specifically, the above-mentioned four parts in the process of TCM diagnosis and treatment are analyzed separately by three analysis methods, including frequency analysis, association rules and hierarchical cluster analysis. And then, a thorough analysis of the whole four parts is conducted using random forest. Considering that the data used in each analysis step have unique characteristics, different analysis schemes are established for different parts of the data.

## Data and methods

### Sample data

The sample data are obtained from the Hospital of Chengdu University of Traditional Chinese Medicine under the confidentiality agreement and the authority approval. According to the Guidelines for the diagnosis and treatment of insomnia in China (2017) [12] and the International Classification of Sleep Disorders(ICSD-3) (2014) [13], the inclusion criteria are set as follows: the medical record data should contain one or more symptoms below: (1) Sleep latency (SL) is prolonged and more than 30 min; (2) Having difficulty in sleep maintenance, mainly manifested by easy and early to wake up; (3) The quality of sleep is decreased, and the patient can hardly get into deep sleep and have multiple dreams; (4) Insufficient sleep duration (less than 6.5 h); (5) With daytime symptoms, including fatigue, emotional problems, memory and attention decline, daytime sleepiness and work initiative decline, etc. The exclusion criteria are set as: (1) The missing of the medical record data is so severe that it is unable to meet the research requirements; (2) The patients have other serious organic diseases that may cause insomnia.

**Fig. 1:**
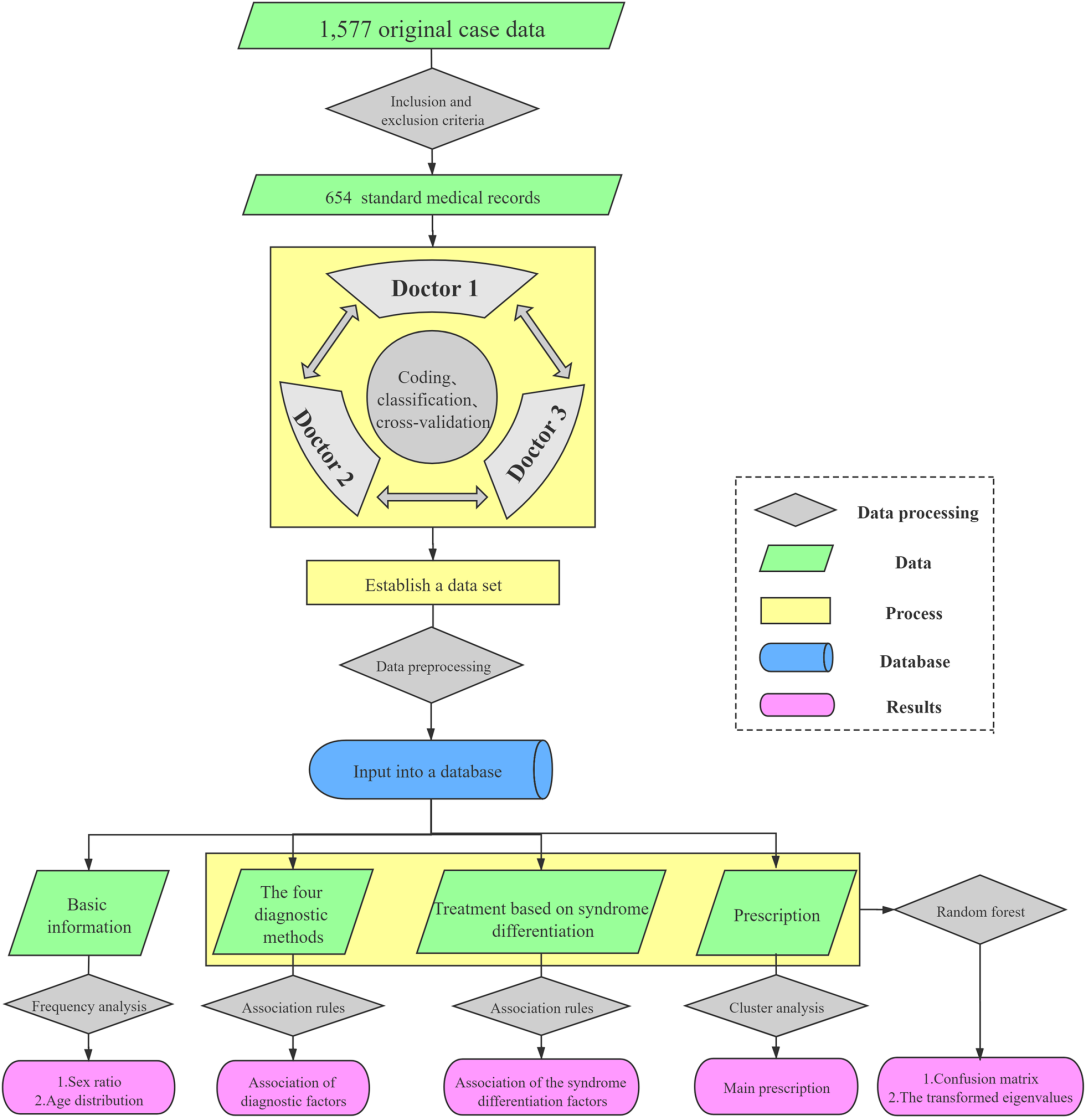
Flowchart of the data processing designed in this paper

In our preliminary work, 1577 outpatient data (from 2016 to 2020) are collected and screened from an experienced doctor of TCM according to the above inclusion and exclusion criteria. The experienced doctor of TCM mentioned here refers to Professor Dongdong Yang. Prof Yang has been devoted to the clinical diagnosis and treatment of insomnia for decades. In the long-term clinical practice, a set of unique TCM diagnosis and treatment system has been formed. Finally, only 654 outpatient data are selected as the research samples. Since the selection and analysis of medical record data of TCM have a high demand for expertise, three professional doctors of TCM (Prof Yang and the other two professional TCM doctors) are selected to analyze, code and classify the medical record data information of research samples manually. Meanwhile, the workload is equally assigned to the three doctors, and the cross-validation is implemented after all work has been completed, so as to eliminate the impact of subjectivity and artificial errors on the final data. Thus, there are only a few differences, and mainly in the treatment based on syndrome differentiation part. For this part, the classifications made by Prof Yang are dominant. Finally, the three doctors would discuss and decide together. And then, the sample database is established. Simultaneously, according to the TCM diagnosis and treatment ideas, the contents of the sample data are divide into four parts: the basic information, the four diagnostic treatment, the treatment based on syndrome differentiation and the main prescription. Each part contains several data, and the specific data processing steps will be described later. In the light of the characteristics of the data, the machine learning methods, including frequency analysis, association rules and hierarchical clustering analysis, are adopted to process and mine the data. Finally, the data of the TCM diagnosis and treatment ideas from the four diagnoses, the treatment based on syndrome differentiation and the main prescription are integrally discussed by employing the random forest algorithm. The specific data processing flow designed in this paper is illustrated in Fig. 1.

The code comparative table is compiled by our research team. In the process of coding and classification, the Guidelines for the diagnosis and treatment of insomnia in China (2017) [12] and the International Classification of Sleep Disorders (ICSD-3) (2014) [13] are regarded as the basis to ensure the objectivity and comprehensiveness of the data. In the meantime, based on the personalized diagnosis and treatment strategy of Prof Yang, a complete code comparative table is shown in Table 1. Three doctors of TCM are required to complete their work in strict accordance with the code comparative table.

**Table 1:** Code comparative table of various insomnia related symptoms

Item	Content	Code	Item	Content	Code	Item	Content	Code
Tongue color	Normal	0	Insomnia course	≤ 3 months	1	Sleep duration	Normal	0
	Pale	1		3 months-1 year	2		Pernoctation	1
	Red	2		1–3 years	3		0–1 h	2
	Dark	3		3–5 years	4		1–2 h	3
	Others	4		> 5 years	5		2–3 h	4
Cold and heat	Normal	0	Asthenia and sthenia	Normal	0		3–4 h	5
	Cold	1		Asthenia	1		4–5 h	6
	Heat	2		Sthenia	2		5–6 h	7
	Cold and heat complex	3		Asthenia and sthenia Complex	3		6–7 h	8
	Others	4		Others	4		> 7 h	9
Five zang-organs	Normal	0	Pathogenic factors	Normal	0		Taking medicine to sleep	A
	Heart	1		Phlegm	1	Emotional status	Normal	0
	Liver	2		Fire	2		Anxious	1
	Spleen	3		Blood stasis	3		Fear	2
	Lung	4		Asthenia	4		Nervous	3
	Kidney	5		Others	5		Restlessness	4
Tongue proper	Normal	0	Sleeping status	Normal	0		Timidity	5
	Enlarged tongue	1		Difficult to fall asleep	1		Irritability	6
	Thin tongue	2		Dysphylaxia	2		Depressed	7
	Teeth print on tongue	3		Festless sleep	3		Flusteredness	8
	Cleft tongue	4		Hard to fall asleep again after waking up	4		Others	9
	The vessels of sublingual purple	5		Dreaminess	5	Concomitant symptoms	Normal	0
	The tongue with ecchymosis							
	Others	6		Others	6		Headache	1
Six fu-organs	Normal	0	Others	Normal	0		Dizziness	2
	Stomach	1		Sallow complexion	1		Lethargic	3
	Gallbladder	2		Flushed cheeks	2		Aversion to cold	4
	Large intestine	3		Redness of the eyes	3		Aversion to heat	5
	Small intestine	4		Dark lip	4		Aversion to cold or heat Irregular	6
	Bladder	5		Halitosis	5		Tidal fever	7
	Sanjiao	6		Eyes bright	6		Night sweat	8
				Others	7		Snoring	9
Pulse conditions	Normal	0	The tongue coating	Normal	0		Nocturia	A
	Thin	1		Yellow	1		Fatigue	B
	Wiry	2		Thin	2		Dry mouth	C
	Slippery	3		Slimy	3		Bitter taste in the mouth	D
	Rapid	4		White	4		Abnormal stool and urine	E
	Deep	5		Scanty	5		Others	F
	Floating	6		Thick	6			
	Others	7		Dye	7			
				Others	8			

### Data processing and machine learning

#### Data preprocessing

Data preprocessing consists of data alignment, missing value processing and data format conversion, etc. It is worth mentioning that the medical record information is extracted strictly according to the coding table, and there are a extremely small number of incomplete cases in the actual medical records. The incomplete items are represented by null values in the process of data set making. To eliminate the impact of the null value on the research and ensure that the follow-up research process can be carried out smoothly, the substitute values are selected to fill the null values of the record data. The substitute values include the course of disease and sleep duration, etc. and these values are filled with their mean value. The substitute values are specified in Table 2.

**Table 2:** Comparative table of the null values and the substitute values

Item	Substitute value
Six fu-organs combinations	Normal
Tongue proper	Normal
Pulse conditions	Normal
Emotional status	Normal
Five zang-organs	Heart
Insomnia course	1–3 years
Asthenia and sthenia	Asthenia and sthenia complex
Cold and heat	Cold and heat complex
Tongue color	Others
Pathogenic factors	Others

The processed data set are import into Python. The data samples are quantified by programming, and then analyzed by applying the following machine learning methods.

#### Frequency analysis

Frequency is also known as "time". The total data are divided into groups according to the preset standards, and then the number of individuals in each group is counted. The relative frequency is the ratio of the frequency of each group to the total number of data.

#### Association rules

A frequently-used method to study the relationship rules among data is to apply the association rules of Apriori algorithm [14]. Generally, three indicators, including confidence, support and lift, can be used to evaluate an association rule. Support is defined as the proportion of the data in the item set to the data in the data set, thus measuring the frequency of a set appearing in the original data. For instance, if two sets in the data set are X and Y respectively, then:

1 $$ Support(X \to Y) = P(X|Y) $$

where X|Y represents the union of X and Y.

Confidence is defined for an association rule. The confidence of X → Y can be expressed as follows:

2 $$ Confidence = {\raise0.7ex\hbox{${p\{ x|y\} }$} \!\mathord{\left/ {\vphantom {{p\{ x|y\} } {P\{ X\} }}}\right.\kern-\nulldelimiterspace} \!\lower0.7ex\hbox{${P\{ X\} }$}} $$

Lift can reflect the correlation between X and Y in association rules. As expressed in the following function, the lift is defined as the proportion of the probability of the data set containing both X and Y to the probability of the data set only containing Y.

3 $$ Lift(X \to Y) = {\raise0.7ex\hbox{${P(Y|X)}$} \!\mathord{\left/ {\vphantom {{P(Y|X)} {P(Y)}}}\right.\kern-\nulldelimiterspace} \!\lower0.7ex\hbox{${P(Y)}$}} $$

The higher the lift is (lift > 1), the higher the positive correlation is, and vice versa. The lift equal to 1 indicates that there is no correlation.

#### Cluster analysis

At present, the cluster analysis is extensively used in the medical field [15]. In general, the cluster analysis can be classified into two categories, one is hierarchical clustering algorithm and the other is agglomerative clustering algorithm. In the Euclidean space, using hierarchical clustering algorithm to analyze small-scale data sets can achieve optimal results. Its basic principle is to establish a hierarchical clustering tree by calculating the similarity among different categories of data points and adopting the bottom-up aggregation strategy. Each sample set in the data sets is regarded as a cluster, and then the clusters with close distance are merged step by step to achieve the expected number of clusters.

Assuming that there are clusters $C_{i}$ and $C_{j}$, the function can be described as follows:

4 $$ D_{aug} (C_{i} ,C_{j} ) = \frac{1}{{|C_{i} ||C_{j} |}}\sum\limits_{{x \in C_{i} }} {\sum\limits_{{z \in C_{j} }} {dist(x,z)} } $$

where the average distance $D_{aug} (C_{i} ,C_{j} )$ is determined by all samples of the two clusters.

#### Random forest

The random forest algorithm derived from ensemble learning method is composed of multiple decision trees. The random forest is an extension of the classification tree and the regression tree. These trees can be used to model the response variables through recursive partition and predict the final results jointly [16]. The random forest algorithm is commonly employed in data classification and regression [17]. At present, there are three mainstream decision tree algorithms, including ID3, C4.5 and CART. In the present paper, the most widely used algorithm, CART, is selected to build random forest algorithm model. The main function of this algorithm is described below.

Suppose that there is a training data set D with k classes in total. The Gini index of set D can be expressed as follows:

5 $$ Gini(D) = \sum\limits_{k} {\frac{{|C_{k} |}}{D}} \left(1 - \frac{{|C_{k} |}}{D}\right) = 1 - \sum\limits_{k} {(\frac{{|C_{k} |}}{|D|})^{2} } $$

where *C*_k_ represents the sample subset of class k. The |*C*_k_| and |*D*| represent the size of *C*_k_ and *D* respectively.

In CART algorithm, assuming that feature A is used to segment the data. If feature A is a discrete feature, set D can be divided into subset D1 and subset D2 according to one possible value a of A, as shown below.

6 $$ D_{1} = \{ D|A = a\} ;D_{2} = \{ D|A \ne a\} $$

Consequently, the *Gini*(*D*,*A*) of set D under the condition of known feature A can be obtained by combining the above functions. The Gini index is theoretically similar to entropy, as described below.

7 $$ Gini(D,A) = \frac{{|D_{1} |}}{|D|}Gini(D_{1} ) + \frac{{|D_{2} |}}{|D|}Gini(D_{2} ) $$

Similar to the principle of entropy, the greater the value of *Gini*(*D*,*A*) is, the greater the sample uncertainty is. Taking this into account, the value of *Gini*(*D*,*A*) should be as small as possible when selecting the feature A.

## Results

### Basic information

**Fig. 2:**
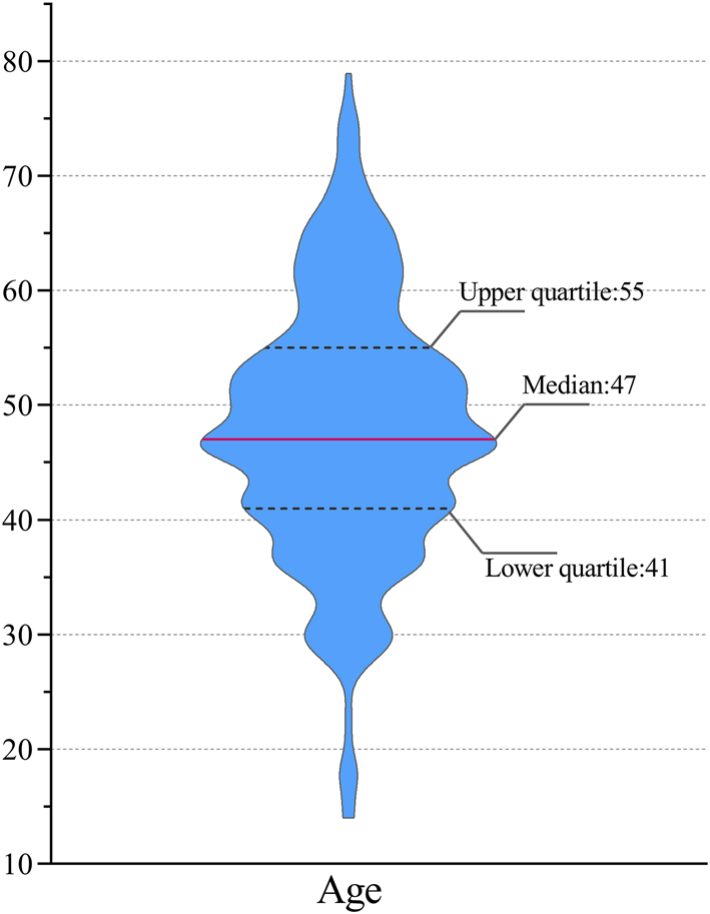
Violin Plot of the age distribution of patients. The average age of patients is 47, the upper quartile of age is 55, the lower quartile of age is 41, and the mean square deviation of age is 11. Moreover, the maximum age and minimum age are 79 and 14 respectively

**Fig. 3:**
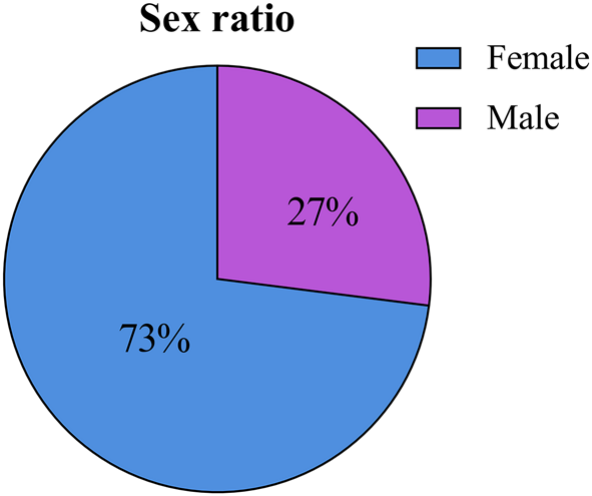
Pie chart of the gender distribution of patients

The basic information mainly consists of the ID, name, clinic time, age and gender of patients. Since the clinic time is not taken as a factor in the screening criteria during the data screening stage, the statistical results may deviate from the actual situation. The ID and name of patients have no impact on the diagnosis and treatment process. As a consequence, the focus of this section is age and gender of patients. Considering that the categories of age and gender data are relatively few, we choose frequency analysis for the data processing. The age distribution of patients shown in Figs. 2 and 3.

### Four diagnostic methods

**Fig. 4:**
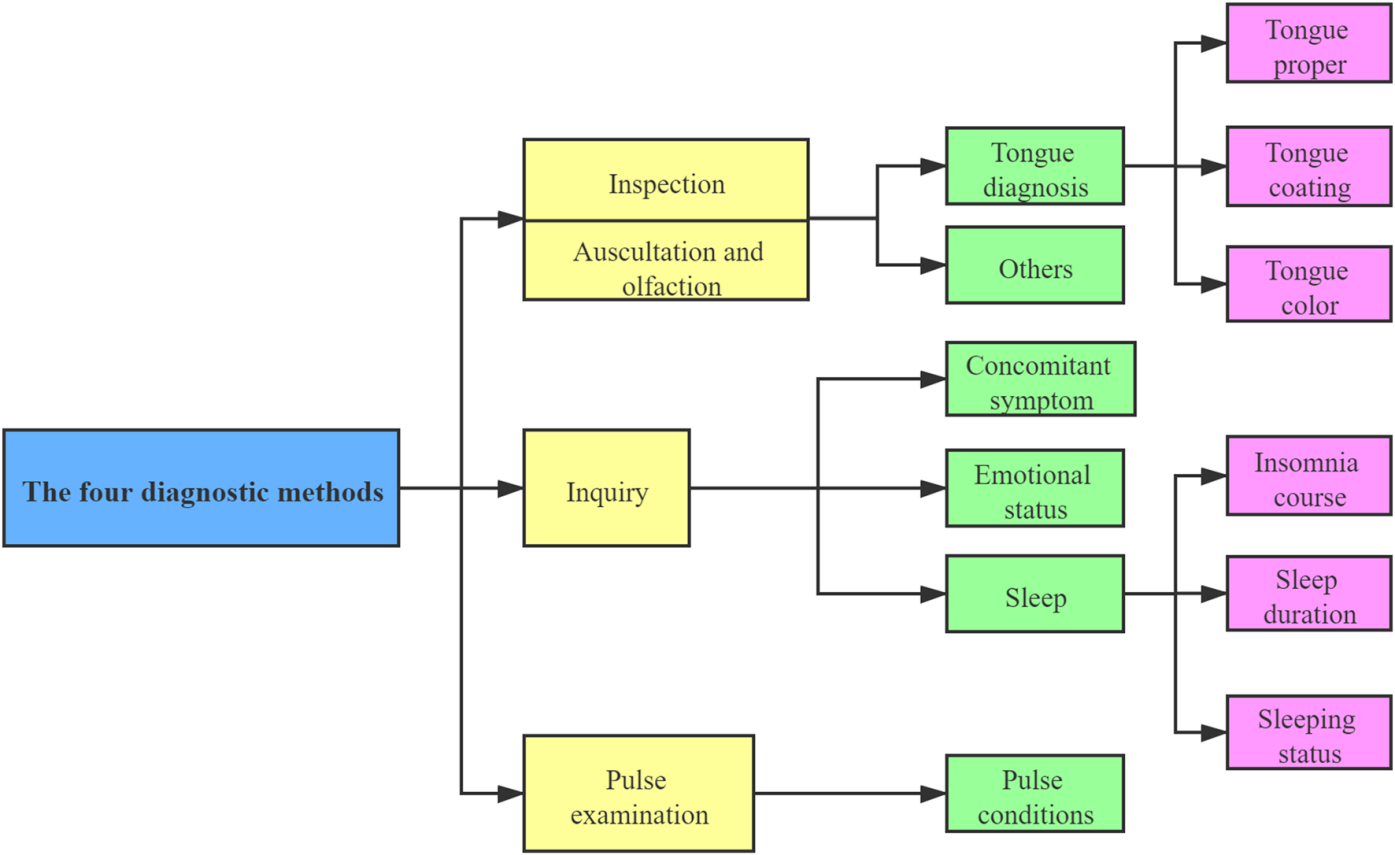
Classification of four diagnostic methods

Four diagnostic methods include inspection (observation), auscultation and olfaction (listening and smelling), interrogation (inquirying or questioning) and palpation (pulse examination). Basically, it is a process of collecting medical history information for doctors of TCM [18]. “Inspection” refers to the observation of patients' external performance, such as tongue picture, expression, reaction and complexion. Moreover, “auscultation and olfaction” is the way that doctors diagnose diseases by hearing and smelling. Additionally, “interrogation” is a sort of diagnostic method for doctors to find out the occurrence, development, treatment process and past health history of diseases by talking with patients. Furthermore, “palpation” particularly refers to the method that doctors use index fingers, middle fingers and ring fingers to touch the special position of radial artery of patients to check the pathological changes of patients. In the process of collecting medical record information through the four diagnostic methods, the amount of information obtained by “inspection” and “auscultation and olfaction” is relatively less than that obtained by “interrogation” and “palpation”. As a result, in the process of data statistics, the “inspection” and “auscultation and olfaction” diagnostic data are combined together for further analysis. Meanwhile, the "tongue diagnosis" data, which is the core of "inspection" diagnosis, are classified and counted separately. In this paper, the four diagnostic methods are further classified on the basis of the characteristics of the medical record data of insomnia research samples (shown in Fig. 4).

Based on the smallest unit of classification, the method of association rules is applied to study in this section. Considering that the basic information is also a part of TCM interrogation and may have an effect on the diagnosis and treatment process of the diseases, the basic information is included in the four diagnostic parts for discussion as well. Taking into account that there are too many null values in some of the smallest classification units, we attempt to use two methods to analyze the association rules for the combinations of the smallest units (the combinations items are in the brackets below), so as to minimize the impact of the null values on the research results. The results are listed in Tables 3 and 4.

**Table 3:** Summary of the results using Method 1

Association	Confidence	Support	Lift
39-year old → Female	1.0	0.02	1.37
59-year old → Female	1.0	0.02	1.37
56-year old → Female	1.0	0.02	1.37
29-year old → Female	1.0	0.02	1.37
(Tongue proper: normal/Tongue color: pale/Tongue coating: thin, yellow/Pulse conditions: thin, wiry) → Female	1.0	0.02	1.37
(Tongue proper: normal/Tongue color: pale/Tongue coating: thin, yellow) → Female	1.0	0.04	1.31
(Tongue proper: teeth print on tongue/Tongue color: red, dark/Tongue coating: thin, yellow) → Female	1.0	0.03	1.3
(Tongue proper: normal/Tongue color: pale, dark/Tongue coating: thin, yellow) → Female	1.0	0.03	1.29
(Sleep duration:normal/Sleeping status:Difficult to fall asleep/Insomnia cours > 5 years) → Female	0.9	0.03	1.29
(Tongue proper: teeth print on tongue/Tongue color: red/Tongue coating: thin, yellow/Pulse conditions: wiry, rapid) → Female	0.9	0.02	1.28
62-year old → Female	0.9	0.02	1.28
50-year old → Female	0.9	0.05	1.28
30-year old → Female	0.9	0.02	1.25

**Table 4:** Summary of the results using Method 2

Association	Confidence	Support	Lift
(Sleep duration:3–4 h /Sleeping status:Difficult to fall asleep/Insomnia course:3 months-1 year) → 47-year old	1.0	0.01	14.22
(Sleep duration:3–4 h /Sleeping status:Difficult to fall asleep/Insomnia course:3 months-1 year) → Male	1.0	0.01	3.72
57-year old → Female	1.0	0.01	1.37
38-year old → Female	1.0	0.01	1.37
39-year old → Female	1.0	0.02	1.37
34-year old → Female	1.0	0.01	1.37
59-year old → Female	1.0	0.02	1.37
56-year old → Female	1.0	0.02	1.37
29-year old → Female	1.0	0.02	1.37
Pulse conditions: thin, wiry, rapid, deep → Female	1.0	0.01	1.37
Pulse conditions: wiry, rapid, deep → Female	1.0	0.01	1.37
(Tongue proper: normal/Tongue color: pale/Tongue coating: thin, white) → Female	1.0	0.01	1.37

Method 1: four diagnostic methods of TCM: (tongue proper, tongue color and tongue coating), (sleep duration, sleep status, course of insomnia, concomitant symptoms and emotion), pulse, age and gender. The results are summarized in Table 3.

Method 2: (tongue proper, tongue color and tongue coating), pulse, (sleep duration, sleep status and course of insomnia), emotion, (concomitant symptoms, others), age and gender. The results are summarized in Table 4.

### Treatment based on syndrome differentiation

Originating from the philosophical culture, the treatment based on syndrome differentiation is the core of the TCM theories and gradually develops into a complex theoretical framework, including the yin and yang theory, five elements, eight principles, the Qi and blood theory, the organs theory and the meridian system [19].

The treatment based on syndrome differentiation is a comprehensive analysis by doctors in the process of diagnosis and treatment of TCM, and its judgment criteria are derived from the objective medical record information including the four diagnoses. The treatment based on syndrome differentiation consists of two processes: syndrome differentiation and treatment. It is not only an essential principle of understanding and treating diseases in TCM, but also a special research and treatment method of diseases in TCM. In the light of the logic of TCM syndrome differentiation, the treatment based on syndrome differentiation can be divided into parts, including the eight principal syndrome differentiation, the organs syndrome differentiation, the meridian syndrome differentiation, etc. Further, these parts can be separated into several items. The previous studies have either skipped this process directly or determined the syndrome differentiation category only based on the experience description of doctors, which were too empirical. Based on the characteristics of insomnia in TCM, this paper focuses on four significant syndrome differentiation points, namely the syndrome differentiation of asthenia and sthenia, the syndrome differentiation of cold and heat, the syndrome differentiation of organs and pathogenic factors. The medical record data are extracted by three professional doctors of TCM, and then classified and coded according to the above four significant syndrome differentiation points. It is worth mentioning that the organs syndrome differentiation includes heart, liver, spleen, lung, kidney, gall bladder, stomach, small intestine, large intestine, bladder and the triple burner; the syndrome differentiation of asthenia and sthenia consists of asthenia syndrome and sthenia syndrome; the syndrome differentiation of cold and heat is composed of cold syndrome and heat syndrome; the pathogenic factors include phlegm, fire, blood stasis and asthenia. The above-mentioned 19 syndrome differentiation factors constitute the section of treatment based on syndrome differentiation of the insomnia sample data research in this paper. To ensure the objectivity of each syndrome differentiation factor, the three TCM doctors are supposed to collect at least two or more kinds of medical record information in the classification and coding stage of medical record data for determining one syndrome differentiation factor. For instance, the medical information "wiry pulse" and "irritability" can infer that the syndrome differentiation factor of organs is liver; the medical information "thin pulse" and "tiredness" can imply that the factor of asthenia and sthenia syndrome differentiation is asthenia syndrome; the medical information "red tongue" combined with "tidal fever" and "rapid pulse " indicates that the factor of cold and heat syndrome differentiation is heat syndrome; the medical information "slippery pulse" combined with "yellow tongue" and "greasy tongue coating" means that the pathogenic factors is phlegm.

Despite that each syndrome differentiation factor in each medical record is relatively independent, there is a strong correlation among the factors. Therefore, it is reasonable to select association rules for the analysis. There are two main reasons affecting the confidence. One is that there are only 654 data selected in this paper, and there are a small number of patients with incomplete medical records, resulting in sparse data distribution. The other is that a variety of classification methods are adopted in this paper, leading to complex classification and more categories of classification combinations. Through a process of trial and error, the confidence is finally adjusted to 0.7 and the results are summarized in Table 5.

**Table 5:** Summary of the results analyzing the syndrome differentiation factors by adopting association rules

Association	Confidence	Support	Lift
Pathogenic factors: fire → Asthenia and sthenia: sthenia	1	0.08	3.86
Pathogenic factors: fire → Cold and heat: heat	1	0.08	1.56
Pathogenic factors: fire/Cold and heat: heat → Asthenia and sthenia: sthenia	1	0.08	3.86
Asthenia and sthenia: sthenia/Pathogenic factors: fire → Cold and heat: heat	1	0.08	1.56
Pathogenic factors: fire** → **Asthenia and sthenia: sthenia/Cold and heat: heat	1	0.08	1.45
Pathogenic factors: fire, blood stasis → Asthenia and sthenia: sthenia	0.96	0.07	3.7
Pathogenic factors: fire, blood stasis → Cold and heat: heat	0.96	0.07	1.49
Pathogenic factors: fire, blood stasis, asthenia → Asthenia and sthenia: asthenia and sthenia complex	0.92	0.08	3.22
Asthenia and sthenia: sthenia → Cold and heat: heat	0.92	0.26	1.43
Pathogenic factors: fire → Five zang-organs: heart, liver	0.89	0.08	2.8
Pathogenic factors: asthenia → Asthenia and sthenia: asthenia	0.89	0.17	1.97
Pathogenic factors: fire, blood stasis → Five zang-organs: heart, liver	0.85	0.07	2.69
Five zang-organs: heart, liver/Six fu-organs: gallbladder → Cold and heat: heat	0.85	0.13	1.33
Five zang-organs: heart, liver → Cold and heat: heat	0.85	0.32	1.32
Cold and heat: normal → Asthenia and sthenia: asthenia	0.82	0.11	1.83
Five zang-organs: heart, liver, spleen/Asthenia and sthenia: asthenia and sthenia complex → Cold and heat: heat	0.82	0.1	1.28
Five zang-organs: heart, liver, spleen/Six fu-organs: normal → Cold and heat: heat	0.8	0.08	1.25
Pathogenic factors: phlegm, asthenia → Asthenia and sthenia: asthenia	0.79	0.09	1.75
Five zang-organs: heart, spleen → Asthenia and sthenia: asthenia	0.76	0.21	1.69
Five zang-organs: heart, spleen/Six fu-organs: normal → Asthenia and sthenia: asthenia	0.75	0.09	1.67
Pathogenic factors: blood stasis, asthenia → Asthenia and sthenia: asthenia	0.73	0.2	1.63
Five zang-organs: heart, liver, spleen → Cold and heat: heat	0.73	0.23	1.14
Asthenia and sthenia: asthenia and sthenia complex → Cold and heat: heat	0.72	0.28	1.12

### Main prescription

Basically, the treatment strategy is composed of acupuncture, moxibustion, scraping therapy and TCM prescription, etc. In the present paper, the TCM prescription is the research focus of this section. TCM prescription is the embodiment of clinical practice of TCM. Choosing the appropriate combinationss of Chinese medicine under the guidance of the treatment based on syndrome differentiation not only reflects the typical thoughts of TCM, but also conforms to the treatment method of drug combinations therapy [20]. Having completed the process from the four diagnoses to the treatment based on syndrome differentiation, the doctors should determine the main prescription. And then, on the basis of the main prescription, the doctors should adjust the prescription properly according to the actual situation of patients. Finally, the treatment prescription can be obtained. Thus, the determination of the main prescription is particularly significant. The main prescription can not only prove the personalized treatment advantages of TCM, but also reflect the most core treatment method in the clinical practice of TCM. The previous studies have achieved some success; however, there are two deficiencies in their researches. First, the previous researches have mainly focused on the frequency of herb use and interrelation of the herbs. Second, there are few previous researches concerned about the components of the main prescription [21, 22]. Taking the above deficiencies into account, the less use herbs are removed from the statistics of the herb use frequency, thus reducing the impact on the research of the main prescription in this paper. Table 6 shows the herbs in the prescriptions through the previous data processing. These codes only represent the corresponding herbs.

**Table 6:** Correspondence between the herbs and the codes

Chinese herbal medicine	Code	Chinese herbal medicine	Code
Spine date seed	1	Pinellia ternate	26
Glycyrrhiza	2	White peony root	27
Anemarrhena	3	Atractylodes Macrocephala	28
Poria cocos	4	Prepared radix rehmanniae	29
Ligusticum wallichii	5	Chinese yam	30
Caulis polygoni multiflori	6	Cornus officinalis	31
Lily	7	Cortex moutan	32
Seed of oriental arborvitae	8	Magnolia officinalis	33
Red peony root	9	Tasteless preserved soybean	34
Gentian	10	Arillus longan	35
Scutellaria	11	Astragalus	36
Gardenia	12	White hyacinth bean	37
Alisma orientale	13	Villous amomum	38
Caulis Aristolochiae Manshuriensis	14	Semen coicis	39
Plantain	15	Os draconis (longgu)	40
Angelica sinensis	16	Oyster	41
Radix rehmanniae	17	Lanceolata	42
Ginseng	18	Radix auckladiae	43
Polygala	19	Placenta hominis	44
Schisandra chinensis	20	Blighted wheat	45
Coptis chinensis	21	Leonurus japonicus	46
Bamboo shavings	22	Cinnamon	47
Citrus aurantium	23	Eucommia	48
Tangerine peel	24	Jianqu	49
Bupleurum	25		

**Fig. 5:**
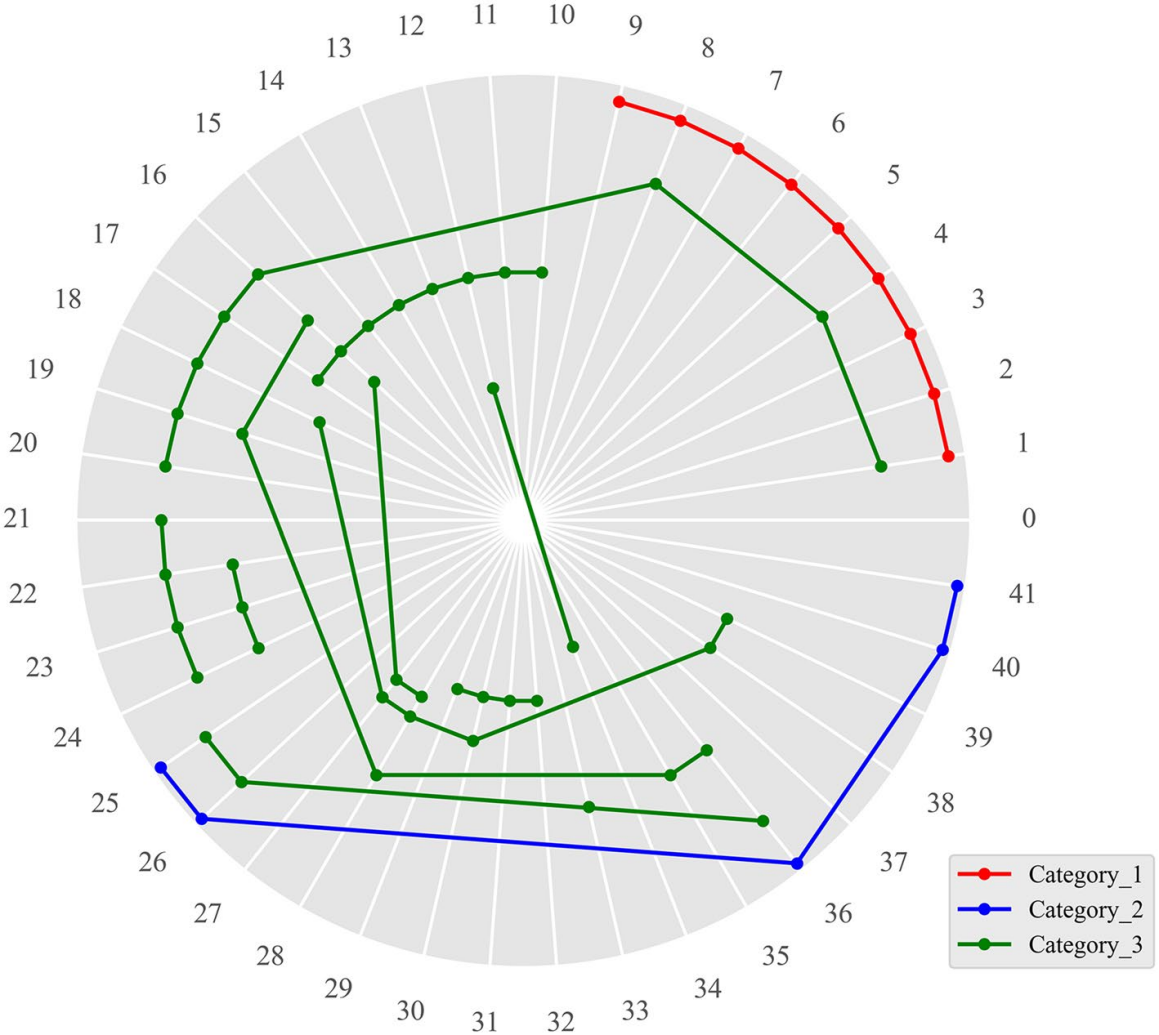
Results of the analysis of the main prescription using hierarchical clustering analysis. The codes in Fig. 5 correspond to the codes in Table 6. The line represents the the main prescription, and the small circle represents the corresponding herb. Frequency of Category 1 is 573, frequency of Category 2 is 312. Frequency of Category 3 is 577, which is the cumulative frequency of all green lines

For the sake of reducing calculation amount and the increasing the code execution efficiency, all the herbs are replaced with codes, and then the codes are entered into the database. The results of the analysis of the main prescription using hierarchical clustering analysis are shown in Fig. 5. The frequencies of the main prescription 3 to 13 are less than that of the main prescription 1 and 2, whose frequencies are 573 and 312 respectively. In order to facilitate comparison and observation, the main prescription 1 (red line in Fig. 5) is determined as Category 1, the main prescription 2 (blue line in Fig. 5) is determined as Category 2, and the sum of frequencies of the main prescription 3 to 13 (green line in Fig. 5) (the sum is 577) is determined as Category 3.

It is necessary to record the prescription information of the medical record data completely and accurately, so all the prescriptions that have appeared repeatedly are counted as the main prescriptions, the main prescriptions of the corresponding serial number are presented in Table 7, and the repeated herb combinations in all main prescriptions are shown in Table 8.

**Table 7:** Correspondence between the main prescriptions and the serial numbers

Serial number	Main prescription	Frequencies
1	Spine date seed, Glycyrrhiza, Anemarrhena, Poria cocos, Ligusticum wallichii, Caulis polygoni multiflori, Lily, Seed of oriental arborvitae, Red peony root	573
2	Bupleurum, Pinellia ternate, Astragalus, Os draconis (longgu), Oyster	312
3	Gentian, Scutellaria, Gardenia, Alisma orientale, Caulis Aristolochiae Manshuriensis, Plantain, Angelica sinensis, Radix rehmanniae	20
4	Angelica sinensis, White peony root, Atractylodes Macrocephala	190
5	Ginseng, Poria cocos, Polygala, Angelica sinensis, Schisandra chinensis, Seed of oriental arborvitae, Radix rehmanniae, Spine date seed	23
6	Coptis chinensis, Bamboo shavings, Citrus aurantium, Tangerine peel	108
7	Bamboo shavings, Citrus aurantium, Tangerine peel	14
8	Bupleurum, Pinellia ternate, Astragalus	108
9	White peony root, Atractylodes Macrocephala, Villous amomum, Ginseng, Chinese yam, Semen coicis	2
10	Atractylodes Macrocephala, Angelica sinensis, Arillus longan, Polygala, Astragalus	30
11	Prepared radix rehmanniae, Chinese yam, Cornus officinalis, Cortex moutan	19
12	Magnolia officinalis	22
13	Tasteless preserved soybean, Gardenia	41

**Table 8:** Correspondence between the repeated herb combinations and the serial numbers

Combinations
Spine date seed, Poria cocos, Seed of oriental arborvitae
Bupleurum, Pinellia ternate, Astragalus
Bamboo shavings, Citrus aurantium, Tangerine peel
White peony root, Atractylodes Macrocephala
Angelica sinensis, Radix rehmanniae
Angelica sinensis, Polygala

### Diagnosis and treatment idea

**Fig. 6:**
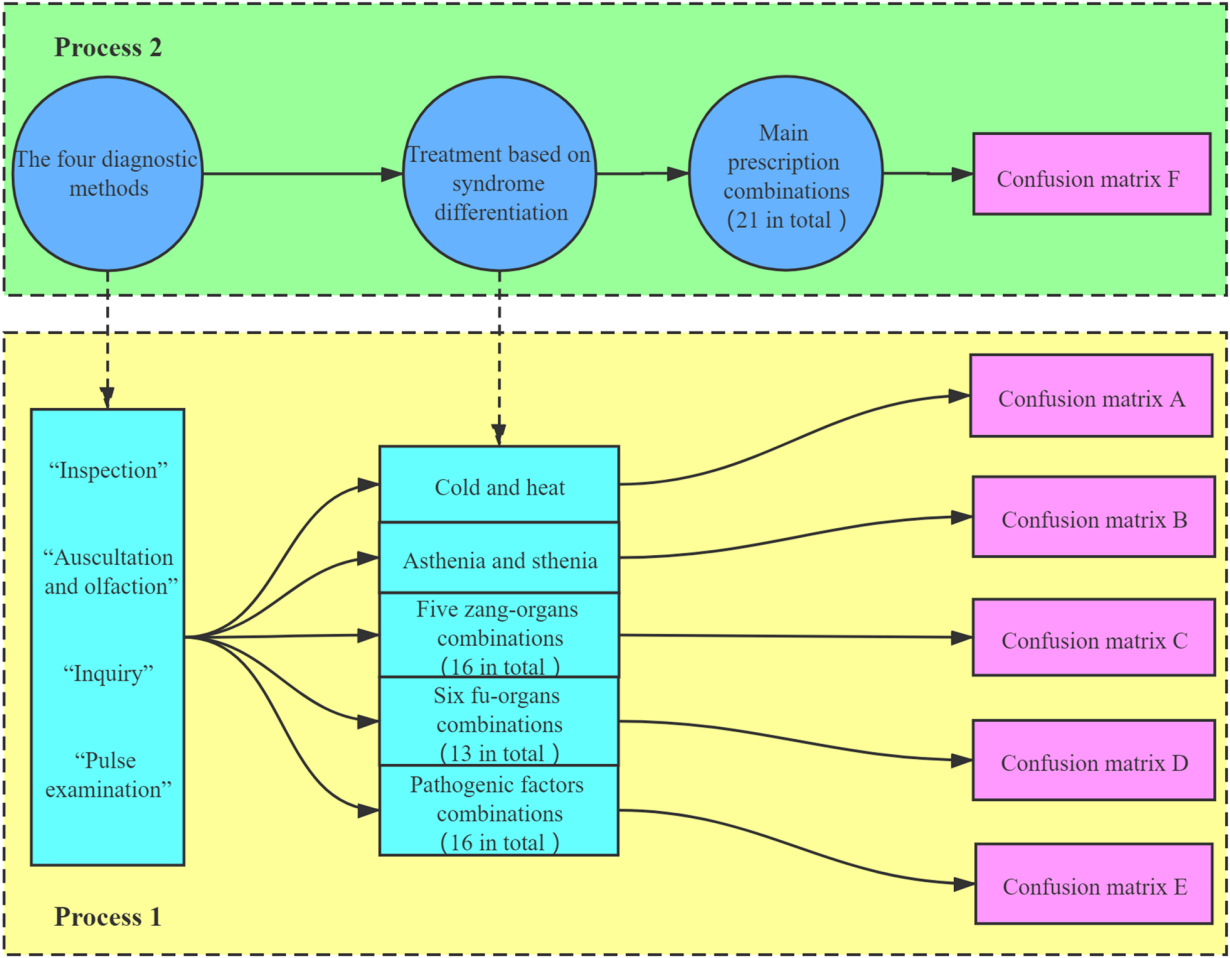
Flowchart of the diagnosis and treatment ideas of TCM. Process 1: the information of the treatment based on syndrome differentiation is deduced from the data of four diagnoses. The information of the treatment based on syndrome differentiation includes five parts: cold and heat, asthenia and sthenia, five zang-organs combinations, six fu-organs combinations and pathogenic factors combinations. Process 2: The main prescription combinations is deduced from the four diagnostic information and the information of the treatment based on syndrome differentiation

In the discussion of the aforementioned four parts, the four parts of TCM diagnosis and treatment ideas are studied successively, so as to reveal the internal relationship and related research methods of each part. This section discusses the four parts as a whole. In accordance with the research process designed in the previous section (in Fig. 1), the random forest algorithm is adopted to establish the model. Simultaneously, the data sets collected from four diagnoses, treatment based on syndrome differentiation, and the main prescriptions of TCM are put into the model for cross-validation by k-fold cross-validation method. Consequently, the corresponding accuracy can be obtained. In the meantime, for the purpose that the internal relationship of TCM diagnosis and treatment ideas can be explored deeper, this section is divided into two processes for further discussion. These two processes are illustrated in Fig. 6.

The data set put into the random forest model includes 654 data. According to flowchart Fig. 6, two processes are designed and six different random forest models are established to realize the prediction of TCM diagnosis and treatment ideas. There are 200 CART decision trees for the models of main prescriptions, and the minimum number of leaf nodes is 5. For the other five models, there are 200 CART decision trees and the minimum number of leaf nodes is 3. The response variables of the six models are cold and heat, asthenia and sthenia, five zang-organs combinations, six fu-organs combinations, pathogenic factors combinations and main prescription combinations.

**Fig. 7:**
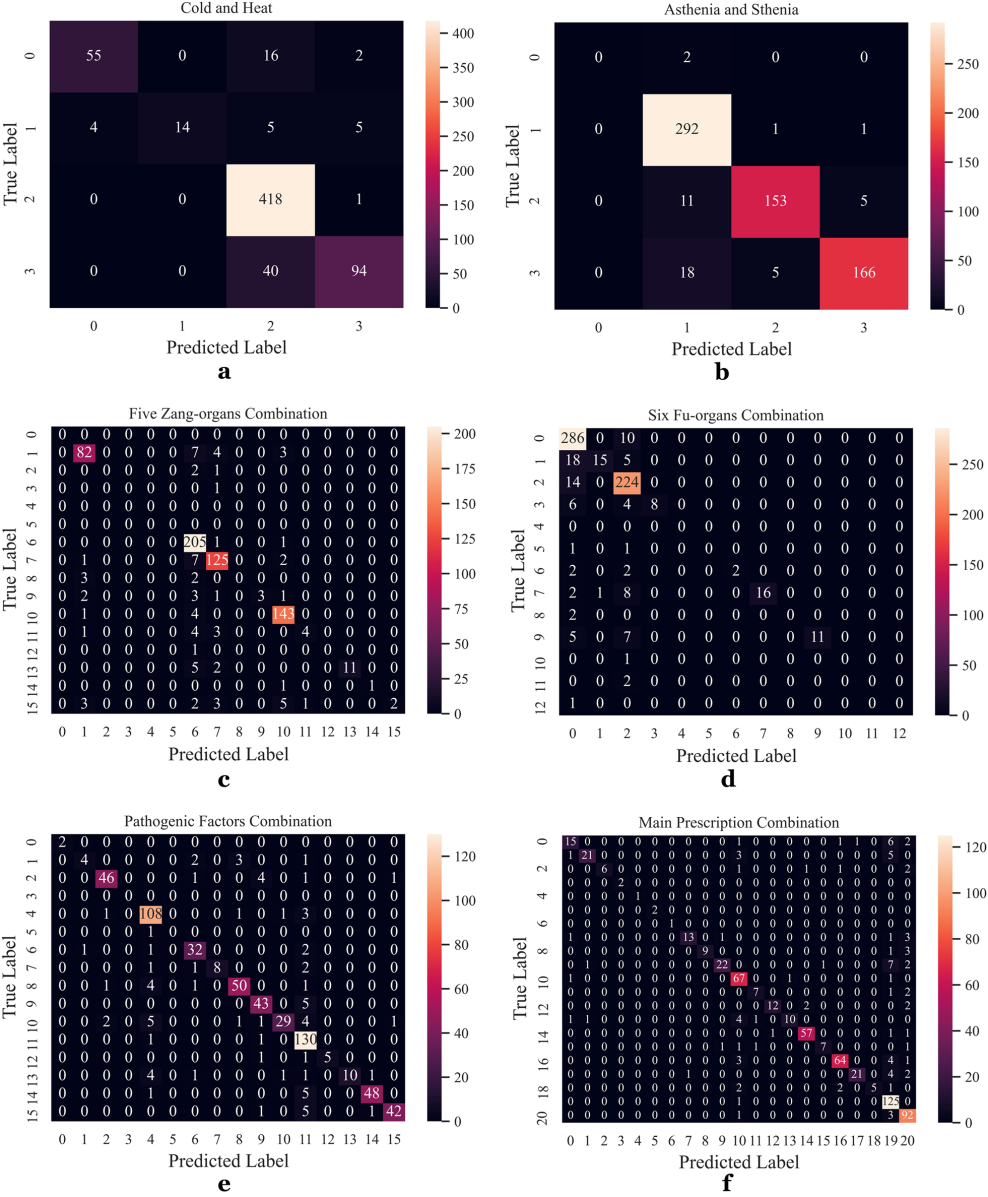
Confusion matrix. Process 1 is shown in **a**–**e**. Process 2 is presented in **f**.In the confusion matrix, the vertical coordinate is the diagnosis made by doctors in the original medical records, and the horizontal coordinate represents the predicted value made by the random forest. The corresponding meanings of independent labels are shown in Tables 1 and 7. Taking the "cold and heat" confusion matrix (as depicted in **a**) in process 1 as an example, the cold and heat syndrome can be derived from the data of four diagnoses. The total number of medical record samples is 654, including 73 cases without cold and heat syndrome, 28 cases with cold syndrome, 419 cases with heat syndrome, and 134 cases with cold and heat complex syndrome. As shown in **a**, among the predicted values of the random forest model, the numbers of the cases accurately predicted by the random forest model for the above syndromes are 55, 14, 418 and 94 respectively. In general, a total of 581 cases are accurately predicted, and the prediction accuracy is 0.89. Similarly, the information of asthenia and sthenia (as depicted in **b**), five zang-organs combinations (as depicted in **c**), six fu-organs combinations (as depicted in Fig. 7(D)), pathogenic factors combinations (as depicted in **e**) and main prescription combinations (as depicted in **f**) can be derived from the the data of four diagnoses, and the numbers of the cases accurately predicted by the random forest model are 611, 576, 562, 557 and 559 respectively

It is worth noting that five zang-organs, six fu-organs and pathogenic factors each contains several syndrome differentiation factors, which are irregularly combined in the actual medical record data. In addition, in the actual outpatient service, the prescriptions made by doctors for patients commonly includes at least one main prescription. Taking the data sample of this paper as an example, 6 independent syndrome differentiation factors labels of the five zang-organs (including a normal item and 5 independent syndrome differentiation factors) can present 16 different combinations labels, while 14 independent main prescriptions labels (including an unprescribed item and 13 independent prescriptions) can present 21 different combinations labels. Therefore, in order to facilitate data processing, the five zang-organs combinations labels, six fu-organs combinations labels, pathogenic factors combinations labels and main prescription combinations labels are coded and loaded into the database. For the sake of presenting the accuracy more intuitively, the method of confusion matrix is carried out in this paper. The confusion matrix results are shown in Fig. 7.

**Fig. 8:**
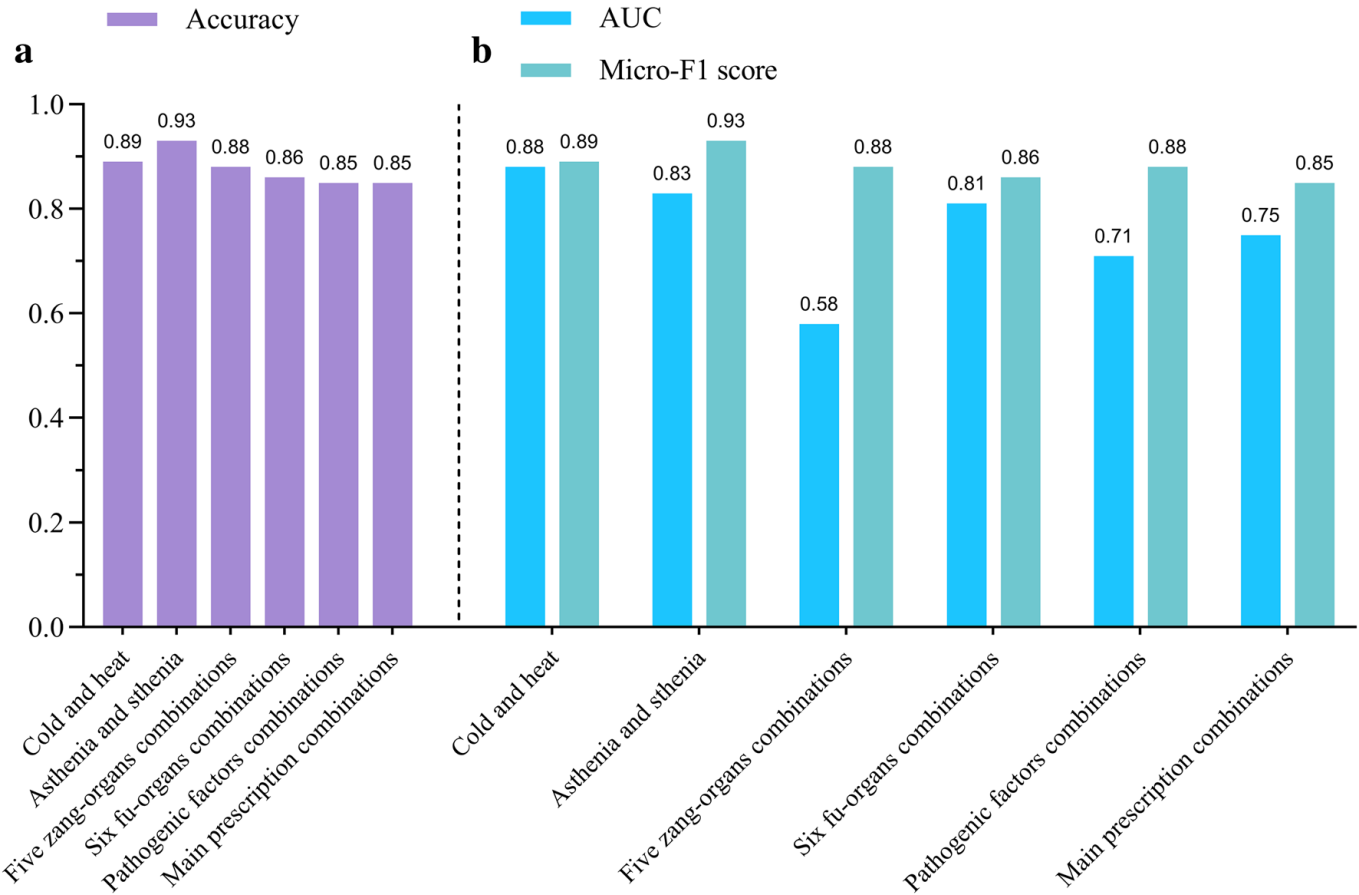
Accuracy, AUC and Micro-F1 score for each model. The accuracy of applying the random forest algorithm models to predict the information of treatment based on syndrome differentiation through the four diagnostic information is shown in **a**. AUC and Micro-F1 score for evaluating the effectiveness and accuracy of the random forest prediction models are shown in **b**

As summarized in Fig. 8a, the accuracy of applying the random forest algorithm model to predict the information of treatment based on syndrome differentiation through the four diagnostic information is dramatically high. Simultaneously, the high accuracy is achieved by predicting the main prescription combinations through the information of the combinations of the four diagnoses and the treatment based on syndrome differentiation.

**Fig. 9:**
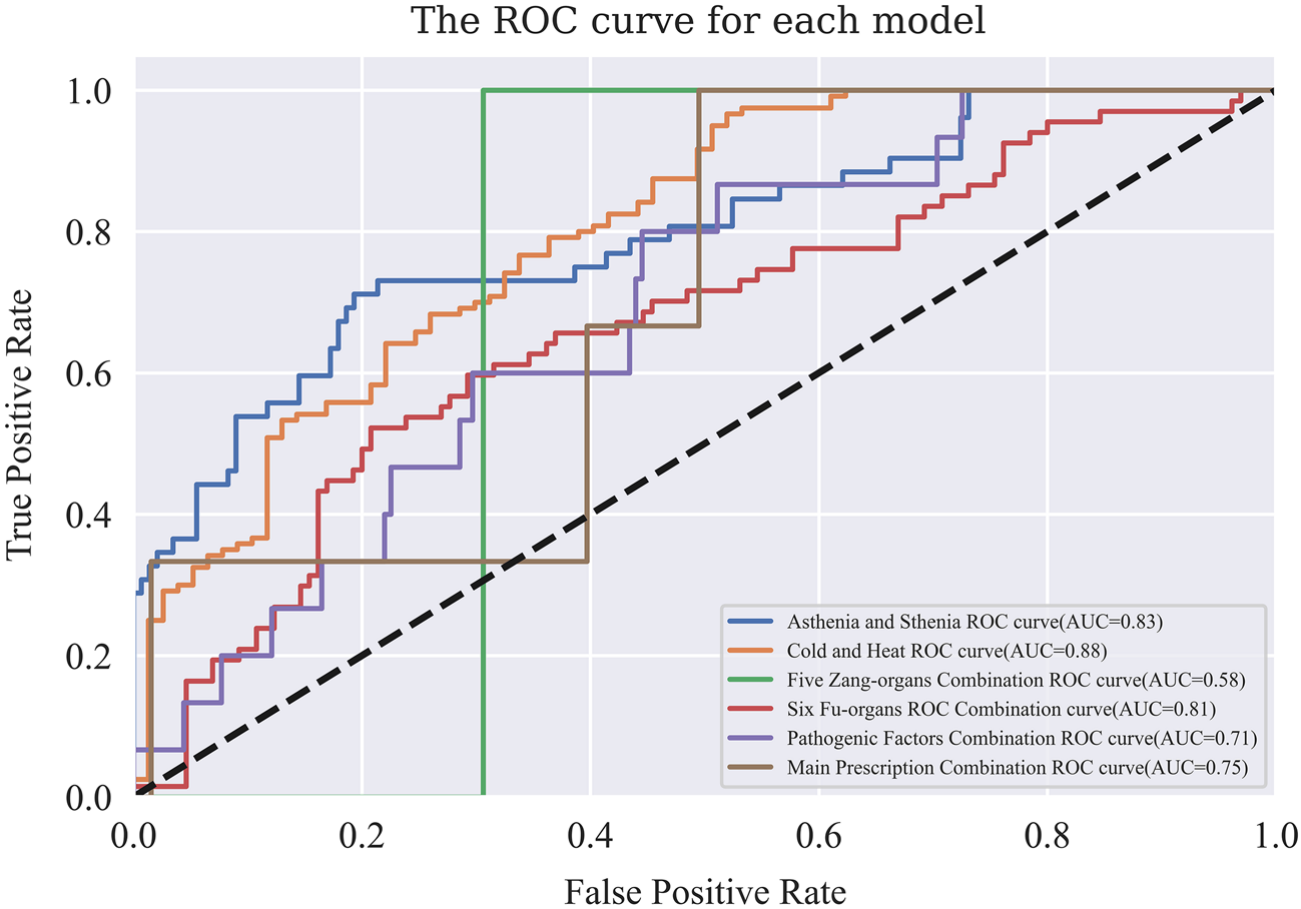
ROC curve for each model. The horizontal coordinate represents false positive rate, and the vertical coordinate represents true positive rate. AUC values of six different models (represented by lines of different colors) established by using random forest algorithm are shown in the legend

The ROC curve is introduced to evaluate the discriminating ability the model’s discriminating ability. As depicted in the Figs. 8b and 9, except for the low AUC value of five zang-organs combinations, the random forest prediction models are effective in verifying the TCM diagnosis and treatment ideas. The false positive rate is higher than true positive rate of the ROC curves of the random forest models of five zang-organs combinations, six fu-organs combinations and the main prescription combinations in some cases. The model of five zang-organs combinations is the most obvious one, leading to the lowest AUC value. There are two reasons for this situation. One is that the amount of sample data used in this study is small. Second, the data are classified by various research strategies, and meanwhile, the data distribution is uneven. The distribution of the five zang-organs data is more uneven than that of other categories data used in other models. The reason for this is that in the process of data coding, the syndrome differentiation of five zang-organs in most clinical insomnia medical records is the heart, followed by the liver, while the number of the syndrome differentiation of spleen, lung and kidney is relatively small. Due to the above reasons, the output of the models is more inclined to the side with higher accuracy in the process of model training. In the random division of the data sets, a small number of samples are divided into the test data sets, thus lacking the training of these samples, resulting in false positive rate [23, 24]. Nonetheless, as the amount of data increases, this problem would be alleviated. Similarly, the models of six fu-organs combinations and the main prescription combinations also show false positive rate in some cases. Since the data distributions are more even, the false positive cases are less than the true positive cases. In our future research, we will improve the efficiency of the models by optimizing the data classification strategies and increasing the amount of data.

The Micro-F1 score is selected to evaluate the accuracy of the models established in this paper. F1 score has been considered as an index used to measure model accuracy in machine learning [25]. Both accuracy and recall of the classification models are taken into account by using F1 score. There are two evaluation indexes for multivariate classification, namely Micro-F1 score and Macro-F1 score. Micro-F1 score is more suitable for unbalanced data distribution. Due to the various classification strategies adopted in this paper and the unbalanced data distribution, Micro-F1 score is selected as the index for evaluating the accuracy of the model. According to the Micro-F1 score (shown in Fig. 8b), it can be seen that the accuracy of each model is dramatically high.

**Fig. 10:**
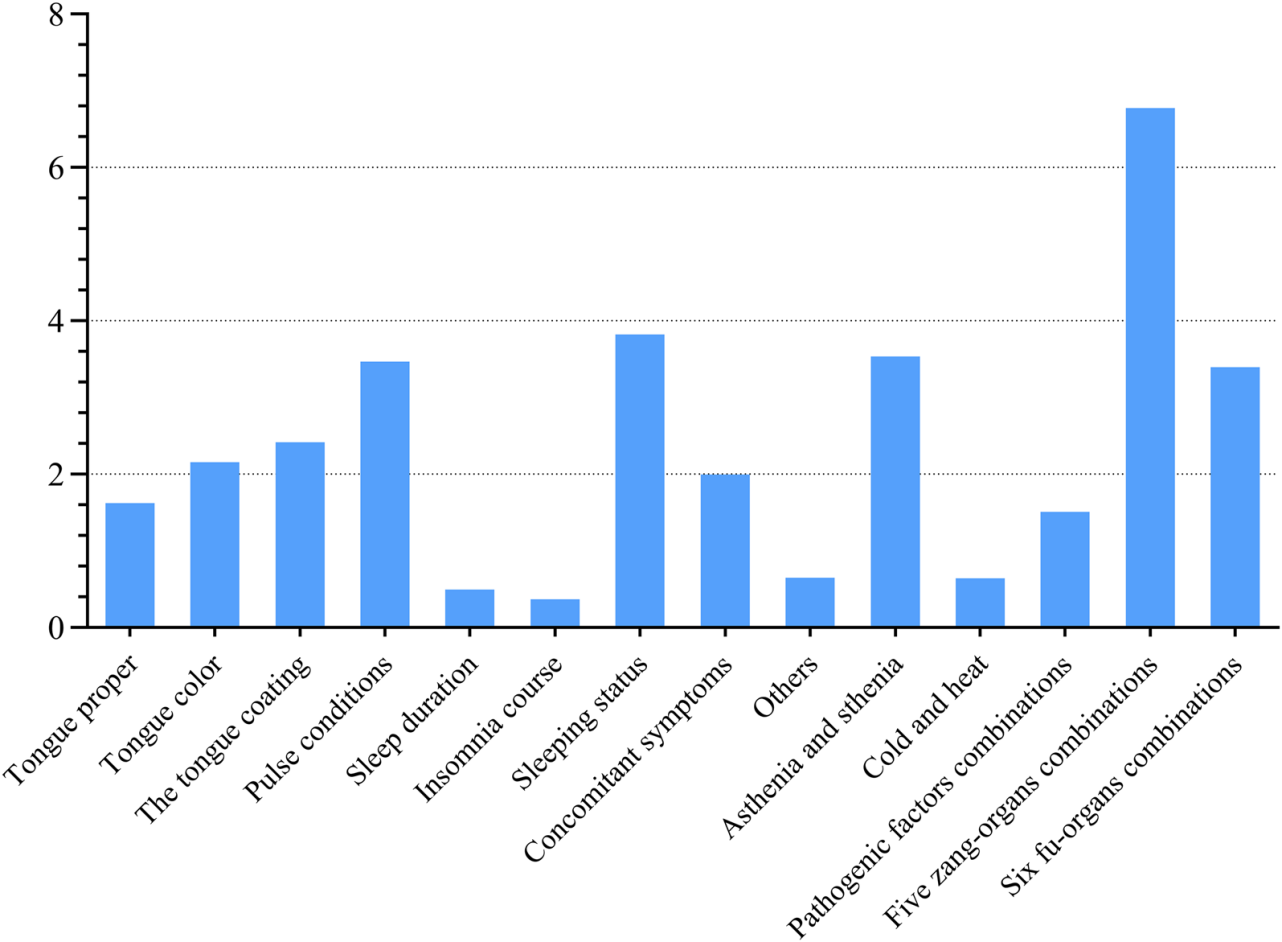
Transformed eigenvalues obtained by using random forest model. The transformed eigenvalues of each input parameter of the random forest model (refers to the process 2 of Fig. 6).

In process 2 of this section, the random forest algorithm model is applied to extract the eigenvalues of all data in the data sets. Since the eigenvalues obtained by using the random forest model are too small to be studied conveniently, the eigenvalues are expanded in the form of logarithmic transformation to facilitate the observation. The transformed eigenvalues are shown in Fig. 10.

## Discussions

**Fig. 11:**
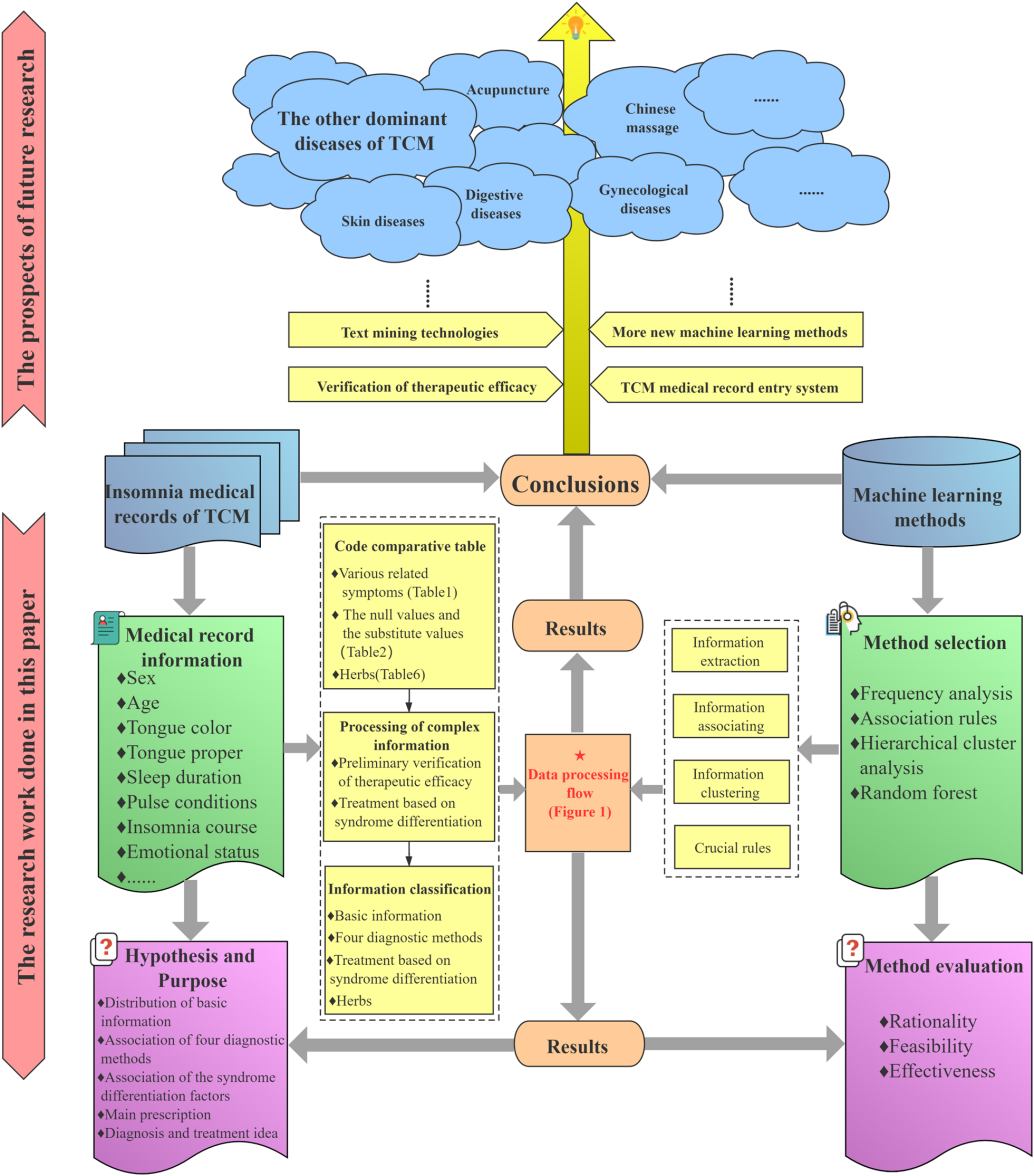
Flowchart of the research strategy designed in this paper

The development of modern medicine mainly has embodied in the continuous improvement of the basic medical theory and clinical practice. In the meantime, the research on the etiology and pathogenic mechanism are more inclined to the micro research. The machine learning methods have been widely used in modern medical related fields [26], such as biochemistry, physiology, microbiology, anatomy, pathology, pharmacology, etc. The concept of wholism and the treatment based on syndrome differentiation are the core principles for diagnosing and treating disease of TCM. The more macroscopic characteristics of diagnosis and treatment also lead to the research of TCM medical record data more complex. Thus, the traditional data analysis methods can hardly be adopted to comprehensively study the diagnosis and treatment process of TCM [6]. Machine learning, as a flexible method for processing complex medical data [27], has been employed in the research of TCM for further progress [28–31]. In the present paper, an innovative research strategy is established to explore the feasibility of machine learning in the study of TCM diagnosis and treatment data. The specific research strategy designed in this paper is illustrated in Fig. 11.

First of all, the frequency analysis is employed to study the basic information of insomnia so that the distribution trend of insomnia in gender and age can be analyzed. As illustrated in Fig. 3, the proportion of female patients is significantly higher than that of male patients. There is evidence that more women than men have insomnia, which is related to the complex interaction of biological, psychological and social factors [32]. The data of insomnia included in this study indicate that insomnia patients are mostly middle-aged women, and meanwhile, middle-aged women are more vulnerable to the influence of perimenopausal syndrome [33], resulting in more female insomnia patients.

Subsequently, the association rules are used to study the data obtained from four diagnostic methods and treatment based on syndrome differentiation. It can be concluded from Tables 3 and 4 that most of the results are dominantly related to gender and age, while there is no significant association among the four diagnoses. Based on these results, it can be found that the information obtained by the four diagnostic methods is complex and relatively independent in the diagnosis process of TCM. Whether the information without any objective connection can play a significant role in the treatment based on syndrome differentiation is the answer to be sought in this paper. At the same time, two innovative research directions can be exploited by concluding the objective results. On the one hand, this research can be explored deeply through expanding the sample size and using other methods to find the internal association of the four diagnoses. On the other hand, there is no obvious external association among the data, but these data have the statistical significance. These data can be used for the epidemiological study of TCM on condition that the sample size is large enough. As can be seen from the Table 5, besides the associations that can be obtained from the basic theories, such as the associations between fire and sthenia syndrome, fire and heat syndrome, there are more new-found associations. For example, the complex syndrome of heart, liver, spleen, asthenia and sthenia → the heat syndrome, the fire stasis syndrome → the heart, liver. The following conclusions can be drawn by analyzing the treatment based on syndrome differentiation with association rules. On the one hand, the results can reveal the connections between complex syndrome differentiation factors and the syndrome differentiation thoughts of TCM doctors. On the other hand, after applying the above methods to classify the contents of treatment based on syndrome differentiation, the results can reflect the priority direction of syndrome differentiation of insomnia to a certain extent, thus having guiding significance for clinical practice. In the further study, more research methods can be adopted to verify the dominant diseases of TCM and explore new syndrome differentiation rules.

Moreover, due to the complexity of the data classification and the small sample size of this paper, the Euclidean distance is selected to evaluate the distance [34]. And the hierarchical clustering algorithm is employed to analyze the small sample data set in the Euclidean distance. According to the characteristics that the main prescription is composed of a wide variety of herbs, the hierarchical clustering algorithm is applied to explore the potential classification rules in the data samples of TCM. There are two purposes of using the hierarchical clustering method to analyze the main prescriptions in this study. One is to obtain the compatibility of the main prescriptions used by the attending doctors from a large number of prescription data. The other is to recode the obtained main prescriptions into the database. The results indicate that the desired purposes can be achieved by adopting the hierarchical clustering algorithm to analyze the main prescriptions. The rapid acquisition of the main prescription of TCM is beneficial for the study of the combinations rules of TCM, but also lays a solid foundation for the overall study of the diagnosis and treatment of the dominant diseases of TCM.

Finally, the random forest method is adopted to discuss the whole diagnosis and treatment process based on the results of the above-mentioned four parts. As illustrated in Fig. 10, the most significant parameter affecting the judgment results is the syndrome differentiation of five zang-organs combinations, followed by sleep status, pulse conditions, the syndrome differentiation of asthenia and sthenia and the syndrome differentiation of six fu-organs combinations. Meanwhile, emotion status, pathogenic factors combinations and tongue picture (including tongue proper, tongue color and tongue coating) also have a tremendous effect on the judgment results. Nevertheless, sleeping duration, insomnia course, syndrome differentiation of cold and heat, and other items except the tongue picture in the inspection and the auscultation and olfaction have less influence on the selection of the final main prescription. As can be seen from the above results, doctors take the sleep status, pulse conditions and tongue picture as the most critical indicators when they are obtaining the four diagnoses information. In the meantime, the emotional status is also taken into account for understanding the basic situation of the patient's condition. Based on the the syndrome differentiation of five zang-organs, and combined with the syndrome differentiation of asthenia and sthenia and the syndrome differentiation of six fu-organs, a comprehensive analysis is conducted to obtain the final main prescription in the process of syndrome differentiation. Since sleep duration, course of insomnia and other factors have little impact on the diagnosis and treatment process, they are only regarded as reference for the diagnosis and treatment. It can be concluded from the above results that the random forest algorithm model can be applied to quickly and accurately verify the correctness of TCM diagnosis and treatment ideas.

In this paper, in order to explore the feasibility of machine learning processing TCM medical record data, the comprehensiveness of outpatient medical record data should be taken into account as the first priority in the data screening phase. On the premise of ensuring the comprehensiveness of the data, due to the complexity of outpatient medical records, such as the incomplete and unquantifiable information contained in the data, the therapeutic efficacy can hardly be verified thoroughly. The preliminary verification of the effectiveness has been carried out by three TCM doctors including the attending doctor Prof Yang in the screening phase of the medical record data. Nonetheless, the verification of the effectiveness has mainly relied on the clinical experience of the three TCM doctors, which may lead to bias on the therapeutic efficacy of single patient with insomnia. Taking the above reasons into account, the actual therapeutic efficacy of each patient has not been fully considered in this study. However, the therapeutic effect is one of the significant evaluation indexes of TCM diagnosis and treatment. In the light of this, it is quite essential to introduce evaluation methods of therapeutic effectiveness in our future research. In the meantime, it is also necessary to further standardize the entry methods of outpatient medical records and establish follow-up records of patients.

Meanwhile, the early outpatient data screening work has been carried out by three TCM doctors, which was extremely time-consuming. In recent studies, various text mining technologies have been applied to processing medical records [35]. In our further research, we will make an effort to employ diverse text mining technologies to extract information, so as to tremendously reduce the waste of resources and improve the efficiency of analysis and processing of TCM medical record data. In addition, the machine learning methods applied in this paper are limited, especially for the whole diagnosis and treatment process, only one algorithm model is used, leading to the lack of diversity of methods. In the future study, we will introduce a variety of algorithm models for comparisons [36–38], and select the optimal model according to the characteristics of different dominant diseases, so as to further study the feasibility of machine learning methods in TCM diagnosis research.

In the future, we will summarize the previous work and establish a simple and easy-to-use TCM medical record entry and analysis system based on machine learning, which will dramatically optimize the process of statistics and analysis of TCM diagnosis and treatment data.

## Conclusions

The results indicate that the machine learning methods can be effectively applied to deeply mine and analyze the medical record data of the dominant diseases of TCM. The focus of this study is to analyze the diagnosis and treatment process of the TCM dominant diseases which includes the acquisition of the patients' condition information through using four diagnostic methods, and the flexible application of the syndrome differentiation methods to develop the treatment plan and select the main prescription. And the normalized research strategy established in this paper can efficiently filter the unessential diagnosis and treatment information, thus helping TCM doctors to quickly and efficiently obtain valuable information and crucial rules from a substantial number of medical record data. In the future, it is essential to establish medical record data entry system, introduce more novel machine learning methods and improve the therapeutic efficacy evaluation of TCM diagnosis and treatment.

## Data Availability

The datasets used and/or analysed during the current study are available from the corresponding author on reasonable request.

## Abbreviations

TCM: Traditional Chinese medicine
CC: Cheng-Church double clustering algorithm
SL: Sleep latency
